# Next-generation sequencing-based bulked segregant analysis without sequencing the parental genomes

**DOI:** 10.1093/g3journal/jkab400

**Published:** 2021-12-04

**Authors:** Jianbo Zhang, Dilip R Panthee

**Affiliations:** Department of Horticultural Science, North Carolina State University, Mountain Horticultural Crops Research and Extension Center, Mills River, NC 28759, USA

**Keywords:** BSA-Seq, PyBSASeq, QTL, genomic region–trait association, structural variants

## Abstract

Genomic regions that control traits of interest can be rapidly identified using BSA-Seq, a technology in which next-generation sequencing is applied to bulked segregant analysis (BSA). We recently developed the significant structural variant method for BSA-Seq data analysis that exhibits higher detection power than standard BSA-Seq analysis methods. Our original algorithm was developed to analyze BSA-Seq data in which genome sequences of one parent served as the reference sequences in genotype calling and, thus, required the availability of high-quality assembled parental genome sequences. Here, we modified the original script to effectively detect the genomic region–trait associations using only bulk genome sequences. We analyzed two public BSA-Seq datasets using our modified method and the standard allele frequency and *G*-statistic methods with and without the aid of the parental genome sequences. Our results demonstrate that the genomic region(s) associated with the trait of interest could be reliably identified via the significant structural variant method without using the parental genome sequences.

## Introduction

Bulked segregant analysis (BSA) was developed for the quick identification of genetic markers associated with a trait of interest (Michelmore *et al.* 1991; Giovannoni *et al.* 1991). For a particular trait, two groups of individuals with contrasting phenotypes are selected from a segregating population. Equal amounts of DNA are pooled from each individual within a group. The pooled DNA samples are then examined via restriction fragment length polymorphism (RFLP) or random amplification of polymorphic DNA (RAPD) analyses. Fragments unique to either group are potential genetic markers that may link to genes that control phenotypic expression to the trait of interest. Candidate markers are further tested against the population to verify the marker–trait associations. With the recent dramatic reductions in cost, next-generation sequencing (NGS) has been applied to more and more BSA studies for the identification of qualitative or quantitative trait loci (QTL; Duveau *et al.* 2014; Clevenger *et al.* 2018; Chen *et al.* 2018b; Arikit *et al.* 2019; Imerovski *et al.* 2019; Lahari *et al.* 2019; Zheng *et al.* 2020). This new technology is referred to as BSA-Seq. In BSA-Seq, pooled DNA samples are not subjected to RFLP/RAPD analysis but are directly sequenced instead. Genome-wide structural variants between bulks, such as single-nucleotide polymorphisms (SNPs) and small insertions/deletions (InDel), are identified based on the sequencing data. Genomic regions linked to the trait-controlling gene(s) are then identified based on the enrichment of the SNP/InDel alleles in those regions in each bulk. The time-consuming and labor-intensive marker development and genetic mapping steps are eliminated in the BSA-Seq method. Moreover, SNPs/InDels can be detected genome-wide via NGS, which allows for the reliable identification of trait-associated genomic regions across the entire genome.

For each SNP/InDel in a BSA-Seq dataset, the base (or oligo in the case of an InDel) that is the same as the reference genome is termed the reference base (REF), while the base that differs from the reference genome is referred to as an alternative base (ALT). Because each bulk contains many individuals, the vast majority of SNP loci in a bulk contain both REF and ALT bases. For each SNP, the number of reads of REF/ALT alleles is termed allele depth (AD). Because of the phenotypic selection via bulking, for trait-associated SNPs, the ALT allele should be enriched in one bulk while the REF allele should be enriched in the other. However, for SNPs unassociated with the trait, both ALT and REF alleles would be randomly segregated in both bulks and enriched in neither. Hence, these four AD values (REF/ALT reads from each bulk) can be used to assess how likely an SNP/InDel is associated with the trait.

QTL-seq is the most widely used software program for BSA-Seq data analysis (Takagi *et al.* 2013; Yamakawa *et al.* 2021). It calculates the REF or ALT allele frequencies of an SNP in both bulks with the four AD values and assesses the likelihood the SNP locus is associated with the trait based on the allele frequency difference (ΔAF) between bulks. Another software program calculates the *G*-statistic value of an SNP with the four AD values and uses the calculated value to judge how likely the SNP is associated with the trait (Magwene *et al.* 2011). We refer to the former as the allele frequency method and the latter as the *G*-statistic method. We have previously developed the significant structural variant method for BSA-Seq data analysis (Zhang and Panthee 2020). In this method, an SNP/InDel is assessed with Fisher’s exact test using the AD values of both bulks. An SNP/InDel with a low *P*-value of Fisher’s exact test tends to have a high absolute ΔAF and *G*-statistic values. An SNP/InDel is considered significant if the *P*-value of Fisher’s exact test is lower than a specific type I error rate, *e.g., α* = 0.01, and nonsignificant if otherwise; a significant SNP (sSNP)/InDel is more likely associated with the trait of interest than a nonsignificant SNP/InDel. Theoretically, sSNPs/InDels should be clustered within and around trait-controlling genes and should not present in genomic regions not associated with the trait of interest. Since SNPs/InDels normally are not evenly distributed across chromosomes, we use the ratio of the significant structural variants to the total structural variants to judge if a genomic region is associated with the trait of interest in our method. This ratio is a parameter at the genomic region level, whereas both the allele frequency and the *G*-statistic value are parameters at the SNP level, which is the key difference between the significant structural variant method and the standard allele frequency and *G*-statistic methods, and the root cause of our method’s higher statistical power in the detection of the genomic region–trait associations.

We tested the significant structural variant method using the BSA-Seq data of a rice cold-tolerance study (Yang *et al.* 2013). One of the parents in this study was rice cultivar *Oryza sativa* ssp. *japonica* cv. Nipponbare. Its high-quality assembled genome sequences were used as the reference sequences for SNP/InDel calling as well, which makes the genotype calling and SNP/InDel filtering very straightforward: any locus in any bulk that is different from the REF allele is a valid SNP/InDel (Zhang and Panthee 2020). Only high-quality assembled genome sequences can serve as the reference sequences in genotype calling, an essential step in BSA-Seq data analysis. For most species, however, such sequences are available for only a single or limited number of lines. If lines without assembled high-quality genome sequences are used as the parents in BSA-Seq studies, the parental genomes are often sequenced via NGS for the determination of the parental origin of SNP alleles and the identification of parental heterozygous SNPs (htSNPs). Modification of our original method to allow the analysis of BSA-Seq data in the absence of assembled or NGS-generated parental genome sequences would provide greater flexibility and significantly reduce sequencing costs. Hence, we modified our original script to allow for the identification of the false-positive SNPs/InDels (homozygous structural variants that are the same in both bulks but different from those in the reference genome) and part of the heterozygous loci in the parents without the aid of the parental genome sequences. Using the modified script and the scripts for the standard *G*-statistic and allele frequency methods (Magwene *et al.* 2011; Takagi *et al.* 2013), we analyzed two public BSA-Seq datasets using either the genome sequences of both the parents and the bulks, or the bulk genome sequences alone. The results revealed that we can only achieve reliable detection of genomic region–trait associations via our modified script when using only the bulk genome sequences.

## Materials and methods

**Figure 1:**
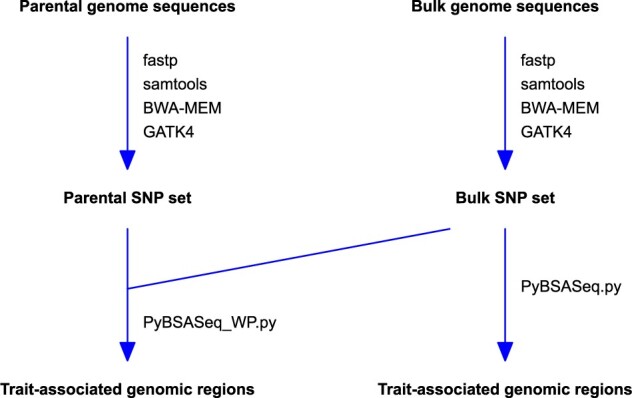
The overview of the analysis workflow. PyBSASeq_WP.py (for the parent and bulk genome sequences) and PyBSASeq.py (for only the bulk genome sequences) are Python scripts for BSA-Seq data analysis.

The rice sequencing data used in this study were generated by Lahari *et al.* (2019). In that study, parents LD24 and VialoneNano were used to develop an F_2_ population of 178 plants. Both the resistant and the susceptible bulk contained 23 plants each. The DNA samples of both the parents and the bulks were sequenced using Illumina MiSeq Sequencing System and MiSeq v3 chemistry. The accession numbers of these sequences are ERR2696318 (parent LD24), ERR2696319 (parent VialoneNano), ERR2696321 (the resistant bulk from the F_2_ population), and ERR2696322 (the susceptible bulk from the F_2_ population). The maize dataset used in this study was generated by Zheng *et al.* (2020). Unlike the rice dataset generated by Yang *et al.* (2013), high-quality assembled genome sequences are not available to the parents of both the rice (Lahari *et al.* 2019) and maize (Zheng *et al.* 2020) datasets used here. However, the parental genomes were sequenced via NGS in both datasets, which is ideal for us to test how parental genome sequences affect the identification of trait-associated genomic regions via the allele frequency, *G*-statistic, and significant structural variant methods. A graphical overview of the analysis workflow is presented in Figure 1.

### SNP calling

The rice BSA-Seq sequencing data were downloaded from the European Nucleotide Archive using the Linux program wget, and the rice reference sequence (Release 47) was downloaded from https://plants.ensembl.org/Oryza_sativa/Info/Index. Sequencing data preprocessing and SNP calling were performed using fastp, samtools, BWA-MEM, and GATK4 (Li *et al.* 2009; McKenna *et al.* 2010; Li 2011, 2013; Chen *et al.* 2018a) as described previously (Zhang and Panthee 2020). When analyzing the BSA-Seq data with the genome sequences of both the parents and the bulks, bulk/parent SNP calling was performed separately. The common SNPs of the two SNP datasets were used for the downstream analysis (Figure 1). The SNP calling-generated .vcf file, but not the raw sequences, is available to the public for the maize dataset. Thus, SNP calling was performed only on the rice sequencing data. For BSA-Seq data analysis in maize, the CHROM, POS, QUAL, REF, ALT, GT, AD, and GQ fields of the .vcf file were extracted to a .tsv file using the GATK4 utility VariantsToTable, and the parental SNP set and the bulk SNP set were created from this .tsv file using a Python script.

### Workflow of the Python scripts

The SNP dataset generated via SNP calling was processed with our Python script to identify sSNP–trait associations. A single script contains all three methods. The workflow of the scripts is as follows:

1. Read the .tsv/.csv input file(s) generated via SNP calling into a pandas DataFrame (size-mutable and potentially heterogeneous two-dimensional tabular data structure with labeled rows and columns; McKinney 2010; Reback *et al.* 2021).
2. Perform SNP filtering on the pandas DataFrame.
3. Calculate the *P*-values of Fisher’s exact test (the significant structural variant method), the ΔAF (allele frequency difference between bulks) values (the allele frequency method), or the *G*-statistic values (the *G*-statistic method) using the four AD values (AD_ref1_ and AD_alt1_ of bulk 1 and AD_ref2_ and AD_alt2_ of bulk 2) of each SNP in the filtered pandas DataFrame. The *P*-values of Fisher’s exact tests, the ΔAF values, and the *G*-statistic values of all SNPs on each chromosome are smoothed by applying a Savitzky–Golay filter (Savitzky and Golay 1964). The smoothed *P*-values are used to identify the sSNPs, while smoothed ΔAF/*G*-statistic values will be used to calculate the corresponding values of sliding windows in step 5.
4. Estimate the threshold of the sSNP/totalSNP ratio, the ΔAF, or the *G*-statistic via simulation. A Savitzky–Golay filter (Savitzky and Golay 1964) is applied to the simulated values of ΔAF and *G*-statistic at the chromosome level as well to smooth the threshold curves. The thresholds will be used to identify the significant peaks/valleys in the plots generated in the next step.
5. Use the sliding window algorithm to plot the sSNP/totalSNP ratios, the ΔAF values, or the *G*-statistic values against their genomic positions.

### SNP filtering

In our previous BSA-Seq study, a parent of the bulks was the *O. japonica* rice cultivar Nipponbare, and its genome sequences were used as the reference sequences for SNP/InDel calling (Zhang and Panthee 2020). In the current dataset, the parents were LD24 and VialoneNano; many false-positive SNPs/InDels and heterozygous loci in the parents would be included in the dataset if analyzing the BSA-Seq data using the original script. Hence, SNP filtering is carried out a little differently from previously described, and its details are below (see Supplementary Table S1 for example):

- Unmapped SNPs or SNPs mapped to the mitochondrial or chloroplast genome;
- SNPs with an “NA” value in any column of the DataFrame;
- SNPs with zero REF read and a single ALT allele in both bulks/parents;
- SNPs with three or more ALT alleles in any bulk/parent;
- SNPs with two ALT alleles and its REF read is not zero in any bulk/parent;
- SNPs in which the bulk/parent genotypes do not agree with the REF/ALT bases;
- SNPs in which the bulk/parent genotypes are not consistent with the AD values;
- SNPs with a genotype quality (GQ) score less than 20 in any bulk;
- SNPs with the sum of its AD values (AD_ref_ + AD_alt_) greater than six times of the average sequencing coverage in any bulk; and
- SNPs heterozygous in any parent when parental genome sequences are available.

In addition, for SNPs with two ALT alleles and zero REF read in both bulks/parents, the REF allele is replaced with the first allele in the “ALT” field, its ALT allele is replaced with the second allele in the original “ALT” field. The REF read and a comma after it are removed from both the AD fields (one for each bulk/parent). This step is carried out before checking the genotype agreement between bulks and the REF/ALT fields. When parental genome sequences are involved, the common SNP set is identified before filtering out the SNPs with a low GQ score in the parental SNP dataset.

### Sliding window settings

The sliding windows algorithm was used to facilitate the visualization of the distribution of the sSNP/totalSNP ratios, the ΔAF values, and the *G*-statistic values across the chromosomes. For all three methods, the size of the sliding windows is 2 Mb and the incremental step is 10 kb in rice; the values of these variables are 5 Mb and 10 kb, respectively, in maize.

### Swapping REF/ALT values of AD and genotype

The tightly linked SNP alleles from the same parent tend to segregate together and should have a similar extent of allele enrichment and thus similar AD values. In an SNP dataset, the genotypes (GT) of each bulk/parent are represented as “GT_ref_/GT_alt_” when an SNP contains both the REF and ALT bases in the GT field and the AD values in each bulk/parent is represented as “AD_ref_, AD_alt_”. The genotype and the AD value of the REF allele are always placed first in both fields. For an SNP locus in the .tsv input file, the allele with the same genotype as the reference genome is defined as the REF allele. However, it is highly unlikely that all of the SNP alleles in a parent are the same as those in the reference genome, except in instances where reference genome sequences used in SNP calling are from one of the parents, as in the case of the cold-tolerance study as mentioned above (Yang *et al.* 2013). It is necessary to place the genotypes and AD values of all SNP alleles from one parent (the reference parent, *e.g.*, LD24) in the REF position, and those from the other parent (*e.g.*, VialoneNano) to the ALT position in the GT and AD fields to make the bulk dataset consistent. Thus, for a particular SNP, if the REF base in the .tsv file is different from the genotype of LD24, its GT/AD values in both bulks would be swapped, *e.g.*, “G/A” to “A/G” and “19,9” (the REF read is 19 while the ALT read is 9 in a bulk in the .tsv input file) to “9,19” (the REF read becomes 9 and the ALT read becomes 19 after AD swapping). AD/GT swapping is performed following SNP filtering and only when the parental genome sequences are used to aid BSA-Seq data analysis. AD swapping ensures that adjacent SNPs have similar ΔAF values. Examples of AD/GT swapping are provided in Supplementary Table S1.

### Calculation of ΔAF/*G*-statistic and estimation of their thresholds

Calculation of the *G*-statistic values and the ΔAF values and estimation of their thresholds were carried out as described previously (Zhang and Panthee 2020). In brief, Equation (1) is used for ΔAF calculation while Equation (2) is used for *G*-statistic calculation. In Equation (1), the first part on the right side of the equation is the ALT allele frequency in bulk 2, while the second part on the same side is the ALT allele frequency in bulk 1. In Equation (2), O is the observed AD (AD_REF1_, AD_ALT1_, AD_REF2_, or AD_ALT2_), *E* is the expected AD under the null hypothesis and is calculated as in the original *G*-statistic method (Magwene *et al.* 2011), and *ln* denotes the natural logarithm. The better the observed values fit the null hypothesis, the closer the expected values are to the observed values and the closer to zero the *G*-statistic value. Most sliding windows contain many SNPs. The ΔAF/*G*-statistic value of a sliding window is the average value of all SNPs in it.

(1) $$AF=\frac{AD_{\mathrm{alt}2}}{AD_{\mathrm{ref}2}+AD_{\mathrm{alt}2}}-\frac{AD_{\mathrm{alt}1}}{AD_{\mathrm{ref}1}+AD_{\mathrm{alt}1}}\#$$

(2) $$G=2\sum_{i}O_{i}\times ln(O_{i}/E_{i})\#$$

Threshold estimation of ΔAF/*G*-statistic is performed for all SNPs in the dataset by simulation. For each SNP in a bulk, its sequencing depth (AD_ref_ + AD_alt_) and the ALT allele frequency in the population are used to simulate its AD_ref_ (smAD_ref_) and AD_alt_ (smAD_alt_) under the null hypothesis. The simulated smAD_ref1_/smAD_alt1_ of bulk 1 and smAD_REF2_/smAD_ALT2_ of bulk 2 are used to calculate the ΔAF or the *G*-statistic. This process is repeated 10,000 times, the 99% confidence interval of the 10,000 ΔAF values is used as a significant threshold for the allele frequency method, and the 99.5th percentile of the 10,000 *G*-statistic values is used as a significant threshold for the *G*-statistic method. As in the real dataset, the threshold of a sliding window is the average value of all SNPs in it.

### Identification of sSNPs and threshold estimation

For each SNP in the dataset, Fisher’s exact test was performed using its four AD values: AD_ref1_/AD_alt1_ of bulk 1 and AD_ref2_/AD_alt2_ of bulk 2. An SNP with its *P*-value less than *α* = 0.01 is defined as a sSNPs. For a trait-associated SNP (located in a trait-controlling gene or tightly linked to the gene), the more the gene contributes to the phenotype, the more its REF/ALT allele would be enriched in either bulk and the more likely it would be identified as an sSNP. SNPs are not distributed evenly along a chromosome, thus it is very likely that different sliding windows contain different numbers of SNPs. Therefore, the sSNP/totalSNP ratio is used to measure sSNP enrichment in a sliding window.

Calculating a threshold requires simulating AD_ref_/AD_alt_ values and calculating the *P*-value of Fisher’s exact test for each SNP in a sliding window. This process needs to be repeated 10,000 times for a sliding window. Doing so for all sliding windows of the SNP dataset would take a very long time. To overcome this obstacle, we first calculate a genome-wide threshold and use it to identify potential significant peaks, then sliding window thresholds of these peaks are calculated via simulation to verify if the sSNP/totalSNP ratios of these peak sliding windows are really significant.

#### Genome-wide threshold

The number of SNPs that are the same as the average number of SNPs per sliding window are randomly selected from the entire SNP dataset. For each SNP in this sample, smAD_ref1_/smAD_alt1_ of bulk 1 and smAD_ref2_/smAD_alt2_ of bulk 2 are obtained via simulation as above. These simulated AD values are used to perform Fisher’s exact test; an SNP with its *P*-value less than *α* = 0.10 is considered an sSNP. This process (starting from sampling SNPs) is repeated 10,000 times, and the 99.5th percentile of these 10,000 simulated sSNP/totalSNP ratios is used as the significance threshold for the detection of potential significant peaks. A higher *α* value (10× higher than that used in the real SNP dataset) is used here, resulting in the identification of more sSNPs from the simulated SNP sub-dataset, hence a higher threshold and fewer false positives.

#### Sliding window threshold

Estimating a sliding window threshold is very similar to estimating the genome-wide threshold; the only difference is that we do not need to sample SNPs from the genome. Instead, smAD_ref_ and smAD_alt_ of each SNP in each bulk in the sliding window are obtained via simulation, and Fisher’s exact test, identification of sSNPs, and sSNP/totalSNP calculation are carried out in the same way as above. This process is repeated 10,000 times, and again the 99.5th of these 10,000 simulated sSNP/totalSNP ratios is used as the threshold for this sliding window.

## Results

We first test how parental genome sequences affected QTL detection in rice. The original sequence reads of the rice data were 3.9G, 3.8G, 3.4G, and 3.5G; they became 3.8G, 3.6G, 3.3G, and 3.4G after quality control, respectively, in ERR2696318 (parent LD24), ERR2696319 (parent VialoneNano), ERR2696321 (the resistant bulk), and ERR2696322 (the susceptible bulk), which correspond to 8.8×, 8.5×, 7.6×, and 7.9× coverage, respectively (Lahari *et al.* 2019). The preprocessed sequences were used for SNP calling to generate an SNP dataset, which was analyzed using the modified significant structural variant method, the *G*-statistic method, and the allele frequency method with or without the aid of the parental genome sequences.

### BSA-Seq data analysis using the genome sequences of both the parents and the bulks

The SNP calling-generated parent/bulk SNP dataset was processed with the Python script PyBSASeq_WP.py. SNP filtering was performed as described in the *Materials and Methods* section. The parental SNP dataset was processed first. All algorithms assume all SNP loci are homozygous in the parental lines, and threshold estimation is based on this assumption. Therefore, the SNPs heterozygous in any parent were eliminated. Although most rice breeding lines should be homozygous in most loci, more than 7% htSNP loci (2,011,062 homozygous and 153,000 heterozygous) were identified in the parental SNP dataset. However, the GATK’s variant calling tools are designed to be very lenient in order to achieve a high degree of sensitivity (https://gatk.broadinstitute.org/hc/en-us/articles/360035535932-Germline-short-variant-discovery-SNPs-Indels-), and it is possible that some sequencing artifacts were identified as heterozygous alleles. The bulk SNP dataset was processed second. The SNPs with the same chromosome ID, the same genomic coordinate, and the same allele composition in both datasets were considered common SNPs. Common SNPs in the bulk dataset were used to detect SNP–trait associations for all three methods.

#### The significant structural variant method

**Figure 2:**
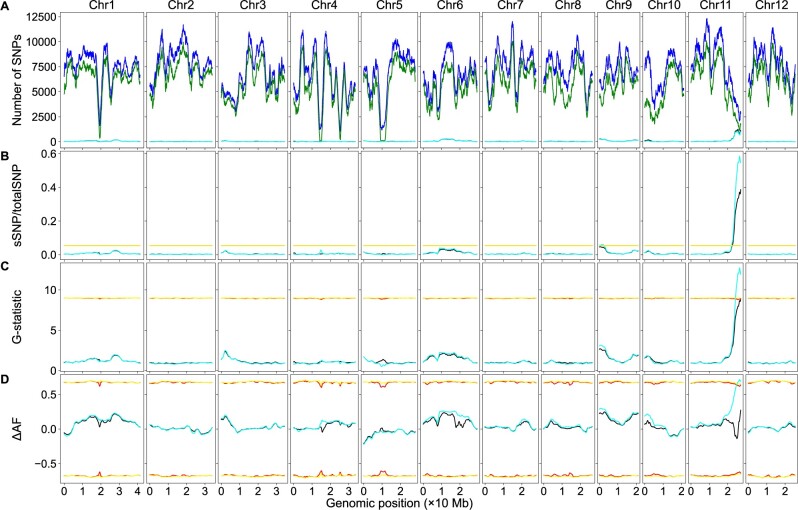
BSA-Seq data analysis using both the parental and bulk genome sequences or the bulk genome sequences alone in rice. The red lines/curves are the thresholds using both the parental and bulk genome sequences, while the yellow lines/curves are the thresholds using only the bulk genome sequences. The black curves represent the number of sSNPs (A), sSNP/totalSNP ratios (B), *G*-statistic values (C), or ΔAF values (D) using the genome sequence of both the parents and bulks. Whereas the cyan curves represent the number of sSNPs (A), sSNP/totalSNP ratios (B), *G*-statistic values (C), or ΔAF values (D) using only the bulk genome sequences. (A) Genomic distributions of sSNPs and total SNPs. Blue curves: total SNPs using genome sequences of both the parents and bulks; green curves: total SNPs using only the bulk genome sequences. (B) Genomic distributions of sSNP/totalSNP ratios. (C) Genomic distributions of *G*-statistic values. (D) Genomic distributions of ΔAF values. Note: due to the value similarity, the cyan curves partially masked the black curves in some genomic regions. The same is true for the yellow lines/curves and the red lines/curves.

Each SNP in the dataset was tested via Fisher’s exact test using its four AD values, and SNPs with *P*-values less than *α* = 0.01 were defined as sSNPs. The chromosomal distributions of the sSNPs and the total SNPs are summarized in Table 1. Using the sliding window algorithm, the genomic distribution of the sSNPs, the total SNPs, and the sSNP/totalSNP ratios of sliding windows were plotted against their genomic position (Figure 2, A and B). A genome-wide threshold was estimated as 0.0538 via simulation, as described in the *Materials and Methods* section. Two peaks above the threshold were identified: a minor one on chromosome 9 and a major one on chromosome 11. The position of the peak on chromosome 9 was at 1.11 Mb, the sliding window contained 230 sSNPs and 3738 total SNPs, corresponding to an sSNP/totalSNP ratio of 0.0615; the position of the peak on chromosome 11 was at 26.44 Mb, the sliding window contained 675 sSNPs and 1139 total SNPs, corresponding to an sSNP/totalSNP ratio of 0.5926. The sliding window-specific threshold was estimated for each peak via simulation, and the values were 0.0551 and 0.0623, respectively, indicating both peaks were significant. Both values are higher than the genome-wide threshold, probably due to the lower amount of total SNPs in these sliding windows. The average SNPs per sliding window was 5893.

**Table 1:** Chromosomal distribution of SNPs—using the genome sequences of both the parents and the bulks

Chromosome	sSNPs	Total SNPs	sSNP/totalSNP
1	1170	139,910	0.0084
2	310	125,129	0.0025
3	459	102,331	0.0045
4	330	89,577	0.0037
5	372	84,706	0.0044
6	1581	83,605	0.0189
7	378	94,371	0.0040
8	258	80,617	0.0032
9	1292	67,157	0.0192
10	363	56,681	0.0064
11	2765	88,287	0.0313
12	241	87,145	0.0028
Genome-wide	9519	1,099,516	0.0087

#### The G-statistic method

The *G*-statistic value of each SNP in the dataset was calculated, and its threshold was estimated via simulation as described in the *Materials and Methods* section. Using the sliding window algorithm, the *G*-statistic value of each sliding window, the average *G*-statistic values of all SNPs in that sliding window, was plotted against its genomic position (Figure 2C), and the curve pattern was very similar to that in Figure 2B. A significant peak was identified on chromosome 11; its position was at 26.43 Mb, its *G*-statistic value was 12.8068, well above the threshold 9.0574.

#### The allele frequency method

The ΔAF value of each SNP in the dataset was calculated, and the ΔAF threshold of the SNP was estimated via simulation as described in the *Materials and Methods* section. Using the sliding window algorithm, the ΔAF value of each sliding window, the average ΔAF values of all SNPs in that sliding window, was plotted against its genomic position (Figure 2D). A significant peak on chromosome 11 was identified, the peak position was located at 26.37 Mb, its ΔAF value was 0.7178, and the 99% confidence interval was to −0.6508 to 0.6508. The ΔAF curves of all chromosomes are very similar to those created by Lahari *et al.* (2019).

### BSA-Seq data analysis using only the bulk genome sequences

The SNP calling-generated bulk SNP dataset was processed with the Python script PyBSASeq.py. The methods and parameters were the same as above; the only difference was that the parental SNP dataset was not used here. Using a different color scheme, the genomic distribution of the sSNPs, the total SNPs, the sSNP/totalSNP ratios, the *G*-statistic values, and the ΔAF values are plotted in Figure 2 for easy comparison.

#### The significant structural variant method

The chromosomal distribution of the sSNPs and total SNPs are summarized in Table 2. The total number of SNPs was 1,346,185 here, much higher than the above, which was 1,099,516. The number of the sSNPs and total SNPs in every sliding window are always higher when the parental genome sequences were not used (Figure 2A). The patterns of the cyan curves (without the aid of the parental genome sequences) were very similar to those of the black curves (with the aid of the parental genome sequences). The sSNP/totalSNP ratios in the low sSNP/totalSNP ratio regions did not change much, and the cyan curves largely overlap with the black curves in these genomic intervals. In contrast, sSNP/totalSNP ratios of the sliding windows were decreased significantly in the high sSNP/totalSNP ratio regions, leading to missing the minor locus on chromosome 9. Only the peak on chromosome 11 was significant; it was located at 26.96 Mb, a 0.52 Mb shift (Figure 2B). The peak sliding window contained 1122 sSNPs and 2945 total SNPs, corresponding to a 0.3810 sSNP/totalSNP ratio, well above the genome-wide threshold (0.0535) and the sliding window-specific threshold (0.0601). The average SNPs per sliding window was 7215.

**Table 2:** Chromosomal distribution of SNPs—using only the bulk genome sequences

Chromosome	sSNPs	Total SNPs	sSNP/totalSNP
1	1,335	163,260	0.0082
2	391	146,877	0.0027
3	578	120,319	0.0048
4	442	110,952	0.0040
5	481	103,362	0.0047
6	1,724	103,416	0.0167
7	459	114,564	0.0040
8	373	103,385	0.0036
9	1,410	82,744	0.0170
10	572	78,206	0.0073
11	3,120	112,719	0.0277
12	281	106,381	0.0026
Genome wide	11,166	1,346,185	0.0083

#### The G-statistic method

The patterns of the *G*-statistic value plot were very similar with or without the aid of the parental genome sequences. Similar to those in the significant structural variant method, the *G*-statistic values were significantly decreased in high-value regions; only a single sliding window was above the threshold (8.8975); its position was at 26.96 Mb, and its *G*-statistic value was 8.9119 (Figure 2C).

#### The allele frequency method

Without the aid of the parental genome sequences, the pattern of the ΔAF curve of chromosome 11, especially the genomic region associated with the trait, was drastically different. Differences in the curve patterns were observed in other chromosomes as well, but they were relatively minor (Figure 2D). No peaks/valleys are significant; all ΔAF values were within the 99% confidence interval, although AD swapping was performed on only 67,396 SNPs, 6.1% of total SNPs.

### Analysis of a maize BSA-Seq dataset

**Figure 3:**
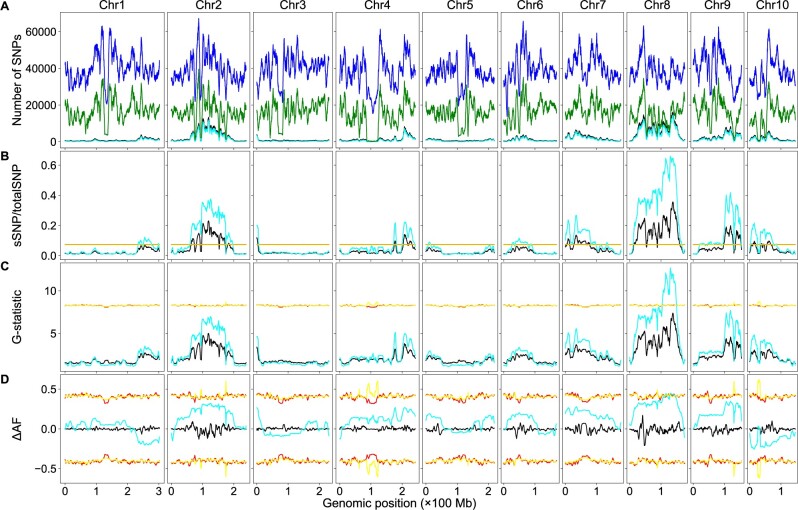
BSA-Seq data analysis using both the parental and bulk genome sequences or the bulk genome sequences alone in maize. The color codes are the same as in Figure 2. (A) Genomic distributions of sSNPs and total SNPs. (B) Genomic distributions of sSNP/totalSNP ratios. (C) Genomic distributions of *G*-statistic values. (D) Genomic distributions of ΔAF values.

Sequencing coverage affects QTL detection. Higher coverage is required to detect minor QTLs than major QTLs (Zhang and Panthee 2020). Rice has the smallest genome size among the major cereal crops; maize has a genome size approximately six times that of rice (Haberer *et al.* 2005). The cost would be much higher in maize than in rice to obtain the same sequencing coverage. Eliminating the need for the parental genome sequences would significantly reduce the sequencing cost for BSA-Seq studies in maize. Here, we analyzed a maize SNP dataset generated by Zheng *et al.* (2020) to test how well our script works on a crop with a much larger genome. Using the dataset, Zheng *et al.* identified a major QTL controlling fertility restoration of the maize *cms-c* (*C-type cytoplasmic male sterility*) gene via the allele frequency method. We analyzed the dataset using the allele frequency, *G*-statistic, and significant structural variant methods. With the aid of the parental genome sequences, results similar to those in Zheng *et al.* were obtained using the allele frequency methods; a significant peak on chromosome 8 was identified. However, many more QTLs were identified using the significant structural variant method: in addition to the major locus on chromosome 8, significant peaks on all chromosomes were identified (Figure 3B), with major QTLs located on chromosomes 2, 8, and 9. Without the aid of the parental genome sequences, the sSNP/totalSNP ratios were decreased significantly in the high-value regions, but significant peaks were still identified on chromosomes 2, 3, 4, 7, 8, 9, and 10. Most peaks are in very similar positions with or without the aid of the parental genome sequences for the significant structure variant methods, but a few peaks shifted significantly. The major peak on chromosome 8 shifted from 129.90 to 138.22 Mb. Most peaks are also in very similar position for the *G*-statistic method, but *G*-statistic values were reduced dramatically without the aid of the parental genome sequences, and no significant peaks were above the thresholds and no QTLs were identified via this method (Figure 3, B and C). However, the ΔAF curves are drastically different across the genome when only the bulk sequences were used and no significant peaks/valleys were detected (Figure 3D). Linked SNPs should have the same *P*-values, ΔAF values, and *G*-statistic values if recombination does not occur between them. Many peaks in a short genomic region on chromosomes 2 and 8 in Figure 3, B–D are observed, which is either curved by high frequency of recombination hot spots in those regions or is just noise caused by other genetic factors or introduced by experiment design. Large duplication could be one of such genetic factors. If a genomic region contains a large duplication and there are many SNPs between the duplicated copies, these SNPs would affect the *P*-values, ΔAF, and *G*-statistic values of sliding windows in this genomic region.

## Discussion

We tested how parental genome sequences affected the detection of SNP–trait associations via BSA-Seq using a dataset of the rice root-knot nematode resistance and a dataset of the maize *cms-c* fertility restoration. We analyzed these datasets with the significant structural variant, the *G*-statistic, and the allele frequency methods. Using the genome sequences of both the parents and bulks, two QTLs in rice and more than 10 QTLs in maize were detected via the significant structural variant method. However, only a single major locus was detected in both maize and rice via the *G*-statistic method or the allele frequency method. The results, including QTL detection, the curve patterns, and peak/valley positions, obtained via the allele frequency method are similar to those in the original studies (Lahari *et al.* 2019; Zheng *et al.* 2020). The positions of the peaks/valleys detected via different methods were not the same, but they were very close to each other (black curves in Figures 2, B–D, and 3, B–D). Using only the bulk genome sequences, most peaks of the sSNP/totalSNP ratios and the *G*-statistic values are in similar positions compared to those with the aid of parental genome sequences, but their values were decreased significantly in many genomic regions, leading to missing some minor QTLs via the structural variant method and all minor QTLs and most of major QTLs via the *G*-statistic method; the only unmissed QTL via the *G*-statistic method has a single sliding window above the threshold (cyan curves in Figures 2, B and C, and 3, B and C). On the other hand, both peak positions and the ΔAF values can be altered dramatically, and no QTL can be detected via the allele frequency method when the parental genome sequences were not used (cyan curves in Figures 2D and 3D).

The significant structural variant method assesses if an SNP is likely associated with the trait via Fisher’s exact test. The greater the ALT proportion differences between the bulks, the less the *P*-value of the Fisher’s exact test, and the more likely the SNP is associated with the trait. Fisher’s exact test takes a numpy array or a Python list as its input, the same *P*-value will be obtained with either [(AD_ref1_, AD_alt1_), (AD_ref2_, AD_alt2_)] or [(AD_alt1_, AD_ref1_), (AD_alt2_, AD_ref2_)] as its input. The *G*-statistic method assesses if an SNP is likely associated with the trait via the *G*-test; the greater the *G*-statistic value of an SNP, the more likely it contributes to the trait phenotype (Magwene *et al.* 2011). The *G*-statistic values are the same with either input [(AD_ref1_, AD_alt1_), (AD_ref2_, AD_alt2_)] or [(AD_alt1_, AD_ref1_), (AD_alt2_, AD_ref2_)]. Changing the AD value (REF/ALT reads) order in the array/list does not affect the *P*-value of Fisher’s exact test or the *G*-statistic value of *G*-test, which is why the parental genome sequences-guided AD swapping does not alter the curve patterns of both methods. Therefore, theoretically, parental genome sequences are not required to identify genomic region–trait associations in either the significant structural variant method or the *G*-statistic method.

When the parental genome sequences were used, AD value swapping was performed for the SNPs in which the genotype of the reference parent was different from the REF base in the reference genome sequences (rice cultivar *Oryza sativa* ssp. *japonica* cv. Nipponbare or maize B73, see the *Materials and Methods* section for details), and the ΔAF values of these SNPs were calculated based on the swapped AD values using Equation (1). AD swapping makes the adjacent SNP loci have similar ΔAF values. The ΔAF values of such SNPs would be calculated using Equation (3) if not performing AD swapping. Equation (3) can be converted to Equation (4), which would produce an opposite value relative to that produced by Equation (1). For two adjacent SNPs in the reference parent, where one SNP has the same genotype as the REF base while the other has the same genotype as the ALT base, they would have opposite ΔAF values if AD swapping is not performed. For the SNPs that do not contribute to the trait phenotype and are not linked to any trait-associated genomic regions, their ΔAF value should fluctuate around zero. The parental genome sequences will have less effect on the ΔAF value of the sliding windows containing such SNPs. However, for trait-associated SNPs, adjacent SNPs with opposite ΔAF values would cancel each other out and lower the absolute ΔAF value of the sliding window significantly. Therefore, parental genome sequences are required to identify genomic region–trait association via the original allele frequency method. The recently published BRM method (Huang *et al.* 2020) is a variant of the allele frequency method. Although it has high detection power due to threshold estimation at the genome level, it is not suitable for BSA-Seq data analysis when the parental genome sequences are unavailable.

(3) $$\Delta \text{AF}=\frac{{\text{AD}}_{\text{ref}2}}{{\text{AD}}_{\text{ref}2}+{\text{AD}}_{\text{alt}2}}-\frac{{\text{AD}}_{\text{ref}1}}{{\text{AD}}_{\text{ref}1}+{\text{AD}}_{\text{alt}1}}$$

(4) $$\Delta \text{AF}=\frac{{\text{AD}}_{\text{alt}1}}{{\text{AD}}_{\text{ref}1}+{\text{AD}}_{\text{alt}1}}-\frac{{\text{AD}}_{\text{alt}2}}{{\text{AD}}_{\text{ref}2}+{\text{AD}}_{\text{alt}2}}$$

We used the bulk SNPs that also exist in the parents for BSA-Seq data analysis when using the genome sequences of both the parents and bulks, and the htSNPs in the parents were filtered out as well. Without the aid of the parental genome sequences, we can only filter out htSNPs containing three or four alleles in an SNP locus (*e.g.*, the genotype of a parent is A/T and the genotype of the other parent is T/G, T/C, A/G, A/C, G/C, GG, or CC at this SNP locus), but not the htSNPs containing only two alleles in an SNP locus (*e.g.*, the genotype of a parent is G/C and the genotype of the other parent is CC, GG, or G/C at this SNP locus). In addition, there are always bulk-specific SNPs (bsSNPs); if a portion of the genome is sequenced in the bulks but not in the parents, the SNPs in this genomic region will be bsSNPs; sequencing artifacts can create bsSNPs as well. Thus, the bulk SNP dataset could contain a significant number of extra SNPs when only the bulk genome sequences were used. In the rice data set, there were 1,345,185 SNPs in the bulk dataset when not using the parental genome sequences, 137,224 of them were htSNPs (heterozygous in the parental lines) while 109,445 SNPs of them were unique to the bulks, and the rest 1,099,516 SNPs were the same as in the dataset with the aid of the parental genome sequences. In the maize dataset, the bulk SNP dataset size was 16,573,961 when only the bulk genome sequences were used, 6,549,258 of them were htSNPs while 3,195,935 of them were bsSNPs, and the rest 6,828,768 were the same as in the dataset with the aid of the parental genome sequences.

**Figure 4:**
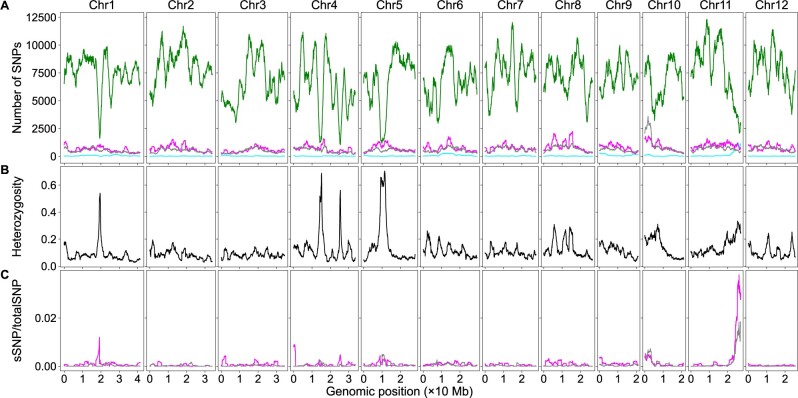
The effects of htSNPs and sequencing bsSNPs on BSA-Seq data analysis when only the bulk genome sequences were used in rice. (A) Genomic distributions of sSNPs (cyan), htSNP (magenta), bulk-specific SNPs (gray), and total SNPs (green). (B) Genomic distributions of SNP heterozygosity in the bulk genome. (C) Genomic distributions of the ratios of sSNPs from htSNPs (magenta) or bsSNPs (gray) to the total SNPs using the dataset with only the bulk genome sequences.

**Figure 5:**
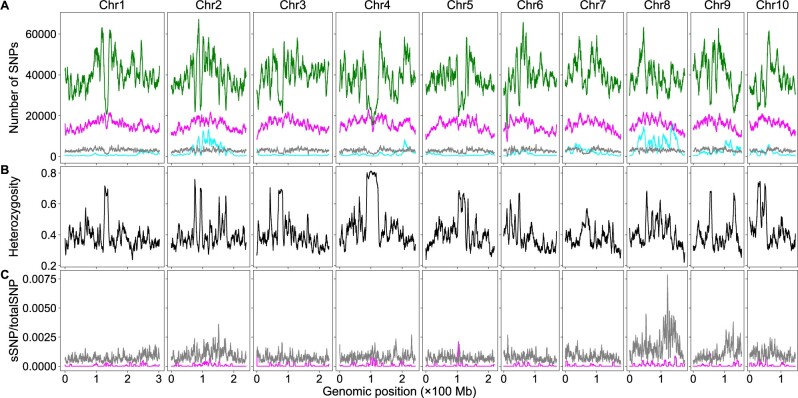
The effects of htSNPs and sequencing bsSNPs on BSA-Seq data analysis when only the bulk genome sequences were used in maize. (A) Genomic distributions of sSNPs (cyan), htSNPs (magenta), bsSNPs (gray), and totalSNPs (green). (B) Genomic distributions of SNP heterozygosity in the bulk genome. (C) Genomic distributions of the ratios of sSNPs from htSNPs (magenta) or bsSNPs (gray) to the totalSNPs using the dataset with only the bulk genome sequences.

To determine how htSNPs and bsSNPs affected the detection of genomic region–trait associations via the significant structural variant method, we extracted the htSNPs/bsSNPs from the bulk SNP dataset and created subdatasets containing only htSNPs or bsSNPs. We first analyzed the rice dataset. We plotted the numbers of htSNPs, bsSNPs, genome heterozygosity, and ratios of sSNPs in htSNPs or bsSNPs to the total SNPs using the sliding window algorithm (Figure 4). Although low at the genome level, heterozygosity can be ∼60% in some SNP sparse regions (Figure 4B). The sSNPs of the htSNPs/bsSNPs contribute little to the overall sSNP/totalSNP ratios in the vast majority of genomic regions, but the sSNPs of the htSNPs contributes ∼0.04 and the sSNPs of the bsSNPs contributes ∼0.02 to the overall sSNP/totalSNP ratios around the major QTL (Figure 4C). The curve patterns of this region in Figures 2B and 4C are similar as well. It is likely that the contribution of htSNPs/bsSNPs to the overall sSNP/totalSNP ratio is real in this genomic region. It is possible that we used too stringent conditions in filtering out htSNPs in the parents so that some identified htSNPs are actually homozygous. Although we cannot rule out the possibility that some bsSNPs were generated by sequencing artifacts, it is possible that some genomic regions were really not sequenced in the parents but were sequenced in the bulks considering the low sequencing coverage (less than 9×). We then plotted the numbers of htSNPs, bsSNPs, genome heterozygosity, and the ratios of sSNPs in htSNPs/bsSNPs to total SNPs of the maize dataset using the sliding window algorithm (Figure 5). The number of bsSNPs is much less than that of the htSNPs (Figure 5A). The genome heterozygosity is ∼39.5% (Figure 5B), indicating an average ∼20% or higher heterozygosity in the parental lines (common htSNPs shared between the parents were counted only once in obtaining the 39.5% heterozygosity rate), which is much higher than that in the rice dataset (∼3.5%). Heterozygosity is very high (>60%) in some genomic regions, and the positions of such regions are correlated very well to the positions of the deep valleys on chromosomes 2 and 8 (Figures 3B and 5B). The maximum contribution of the sSNPs in htSNPs/bsSNPs to the overall sSNP/totalSNP is less than 0.0075. Thus, both htSNPs and bsSNPs mainly increase the total SNPs, not the number of sSNPs, which significantly decreases the sSNP/totalSNP ratios in the QTL regions. Low genome heterozygosity as in the rice dataset has very minor effects on the peak position shift, but too many htSNPs as in the maize dataset can significantly shift some peaks of sSNP/totalSNP ratios and *G*-statistic values. It is difficult to remove htSNPs with two alleles from the dataset in a software approach without the aid of parental genome sequences, but their negative effects on the sSNP/totalSNP ratios and peak shift can be minimized if F_2_ populations are constructed with seeds from a single F_1_ plant or the parents are selfed more generations.

All three methods can be used to identify both qualitative and QTL; the allele frequency is particularly informative to determine if an SNP is associated with a qualitative or quantitative trait locus. For an incomplete dominant locus, we expect 100% of ALT allele frequency in one bulk while 0% of ALT allele frequency in the other bulk. For a dominant locus, one bulk should contain 66.7% heterozygous and 33.3% dominant homozygous individuals, corresponding to 66.7% ALT allele frequency (treat the dominant allele as the ALT allele), and the other bulk should contain 100% recessive homozygous individuals, corresponding to 0% ALT allele frequency. For a QTL, allele enrichment is dependent on how much it contributes to the trait. The major QTL would have high allele enrichment, while the minor QTL would have low allele enrichment. The ALT allele frequency of an SNP in a QTL should be lower than 100% but could be higher than that of a dominant locus. The peak on chromosome 11 in rice (Figure 2B) should represent an incomplete dominant locus: a cluster of SNPs around the peak have allele frequency of 100% in one bulk and 0% in the other bulk. The highest peak on chromosome 8 in maize (Figure 3B) is very likely a dominant locus: a cluster of SNPs around this peak have allele frequency of ∼67% in one bulk and 0% in the other bulk. The highest frequency of such SNPs is around 140 Mb on chromosome 8, very close to the peak identified with only the bulk genome sequences. As expected, the SNP alleles with 0% allele frequency in the cluster are from the same bulk (after AD/GT swapping and removing htSNPs and bsSNPs) in both rice or maize. Some SNPs with ALT allele frequency not equal to 0% intermingled in the clusters was observed in these regions; it is very likely they are caused by sequencing artifacts or SNPs between large duplications in the genome. The other significant peaks should represent QTLs. Our results suggest that rice nematode resistance is controlled by a single incomplete dominant gene with a minor modifier while maize *cms-c* fertility restoration is controlled by a dominant gene with many modifiers. These modifiers are most likely QTLs.

Both the allele frequency and the *G*-statistic methods use an SNP level parameter to identify significant sliding windows to detect the genomic region–trait associations. The significant structural variant method, however, uses the sSNP/totalSNP ratio, a parameter at the sliding window level, to measure the sSNP enrichment in a sliding window for the identification of the trait-associated genomic regions. An SNP normally has less than 100 reads because of the cost concern, while a sliding window normally contains thousands of SNPs. Thus, the significant structural variant method has much higher statistical power, which is consistent with our observations. Moreover, we used *α* = 0.01 for the identification of sSNPs in the real SNP dataset, whereas we used *α* = 0.1 for the same purpose in the simulated SNP dataset when estimating thresholds, leading to the identification of more sSNPs in the simulated SNP dataset and thus higher sSNP/totalSNP thresholds. For the rice dataset, the genome-wide threshold is 0.0538 with the aid of the parental genome sequences and 0.0535 without the aid of the parental genome sequences with *α* = 0.1 for threshold estimation (Figure 2B). When using *α* = 0.05 for threshold estimation, the thresholds are much lower, 0.0275 with the aid of the parental genome sequences and 0.0273 without the aid of the parental genome sequence; in addition to the major locus on chromosome 11 and the minor locus on chromosome 9, a significant peak on chromosome 6 was identified with or without the aid of the parental genome sequences. Similarly, the sSNP/totalSNP thresholds were decreased significantly in maize using *α* = 0.05 for threshold estimation and thus more minor QTLs were detected. The sSNP/totalSNP ratio of a sliding window within a genomic region not associated with the trait should be zero. The sSNP/totalSNP ratios of the sliding windows on the entire chromosomes 2, 7, 8, and 12 and the vast majority of the genomic regions of the other chromosomes are very close to zero in Figure 2B, and the ratios of the majority regions of chromosomes 1, 3, and 5 in Figure 3B are very close to zero as well, suggesting the background noise of the significant structural method is very low and it is reasonable to use *α* = 0.05 for threshold estimation. Therefore, the significant structural variant method can be used to reliably detect major QTLs and some minor QTLs when the parental genome sequences are not available.

## Data availability

The scripts and their usage can be found on https://github.com/dblhlx/PyBSASeq. The rice raw BSA-Seq sequencing data are available on https://www.ebi.ac.uk/ena/browser/view/PRJEB27629. Small subsets of the rice and maize parental/bulk SNP/InDel datasets used in this study are deposited on https://github.com/dblhlx/PyBSASeq/tree/master/Data and can be used as the input file(s) to test the Python scripts.

Supplementary material is available at *G3* online.

## Supplementary Material

jkab400_Supplementary_Data
